# Return To Sports After Talar OsteoPeriostic Grafting From the Iliac Crest for Large Osteochondral Lesions of the Talus

**DOI:** 10.1177/23259671261418058

**Published:** 2026-03-26

**Authors:** Jari Dahmen, Elze J. Geurts, Quinten Rikken, Sjoerd A.S. Stufkens, Gino M.M.J. Kerkhoffs

**Affiliations:** † Department of Orthopedic Surgery and Sports Medicine, Amsterdam University Medical Centers, Amsterdam, North Holland, the Netherlands; Investigation performed at Department of Orthopedic Surgery and Sports Medicine, Amsterdam University Medical Centers, Amsterdam, North Holland, the Netherlands

**Keywords:** OLT, cartilage, ankle, TOPIC, return to sport

## Abstract

**Background:**

The Talar OsteoPeriostic grafting from the Iliac Crest (TOPIC) procedure shows successful clinical outcomes up to 2-year follow-up for large osteochondral lesions of the talus (OLTs). However, sports outcomes remain unknown.

**Purpose/Hypothesis:**

The purpose of this study was to prospectively evaluate sport outcomes for patients with large OLTs treated with the TOPIC procedure. It was hypothesized that the TOPIC procedure would result in adequate (return to/participation in) sports outcomes and improvements of pain scores during running at 1 and 2 years after surgery.

**Study Design:**

Cohort study; Level of evidence, 3.

**Methods:**

A total of 61 patients who underwent the TOPIC procedure for a symptomatic OLT were prospectively included with ≥24-month follow-up. Using digital questionnaires and an in-depth qualitative telephone interview, participation in desired level of sport at 1 and 2 years (primary outcome) was assessed. Secondary outcomes included different levels of return to and participation in sports rates, as well as the Numeric Rating Scale (NRS) for pain during running and the Ankle Activity Score (AAS) at 1 and 2 years postoperatively.

**Results:**

Of the total patients, 59% and 82% participated at their desired level of sport at 1 and 2 years after TOPIC, respectively. Furthermore, at 2 years postoperatively, the return to any level of sports was 98%, the return to preoperative level of sports was 93%, and the return to preinjury level of sports was 59%. The AAS significantly increased from 2 (IQR, 2-3) preoperatively to 5 (IQR, 5-6.5), 2 years postoperatively (*P* < .01). The NRS for pain during running improved significantly from 9 (IQR, 8-10) points preoperatively to 5 (IQR, 1-8) points at 2 years postoperatively (*P* < .01). At 2-year follow-up, the reoperation rate was 38%.

**Conclusion:**

Almost all patients returned to sports, and 8 out of 10 returned to their desired level of sport 2 years after the TOPIC procedure. Ankle activity significantly improved, and pain during running significantly decreased. Moderate symptoms, mostly unrelated to the graft, persisted in some patients, and 1 in 3 required reoperation.

Ankle injuries, such as ankle sprains and fractures, are among the most common sports injuries.^4,10^ Such injuries may lead to cartilage damage in the ankle in the form of an osteochondral lesion of the talus (OLT).^5^ Patients with OLT often experience deep ankle pain during or after weightbearing activities, which can substantially limit their level of physical activity and participation in sports.^9^

Currently, no consensus regarding the optimal treatment for OLT has been reached.^6,16,23,29^ OLTs vary greatly in clinical presentation and radiological morphology. This means that treatment is individualized and depends on previous treatment, size, morphology, and location.^21^ Large primary and nonprimary osteochondral lesions (>15 mm in diameter) of the talus are often treated with an autograft or allograft transplantation in which the damaged osteochondral unit is replaced.^26,28^ Osteochondral autograft procedures allow for adequate clinical outcomes and return to sport (RTS) rates.^6,16,24^ However, a disadvantage of autologous osteochondral transplantation is harvesting the graft from the ipsilateral femoral condyle, which can lead to donor-site morbidity in up to 32% of patients, potentially affecting their ability to return to or participate in sport.^6,14^ To avoid donor-site morbidity, a new joint-sparing technique for large, primary, and nonprimary OLTs was created; the Talar OsteoPeriostic grafting from the Iliac Crest (TOPIC) procedure.^15^ Although the TOPIC procedure showed satisfactory clinical outcomes at up to 2 years of follow-up, it is currently unknown what the sports outcomes are for this group of patients who have undergone this relatively new surgical treatment option.^8^ These results are important, as sports can have a substantial impact on quality of life, especially in patients with an athletic background before injury.

As such, the primary aim of this study is to assess the participation in the desired level of sport rate 1 and 2 years after the TOPIC procedure for OLT. The secondary aims were to assess different levels of RTS, level of participation in sports, and sports-related patient-reported pain and functional outcomes at 1 and 2 years after the TOPIC procedure. Our hypotheses were that the TOPIC procedure for large OLTs would result in adequate (return to) sport outcomes and improvements in sports-related patient-reported outcomes 1 and 2 years after the surgery.

## Methods

The present study is a prospective, nonrandomized, single-center, clinical comparative cohort study. The study was performed in accordance with the current ethical standards (Declaration of Helsinki) and is an accredited center for the diagnosis and treatment of cartilage and osteochondral lesions of the foot and ankle.

### Patient Selection

The study included all patients with a symptomatic OLT who were treated with the TOPIC procedure and had a minimum follow-up of 24 months. The TOPIC procedure is indicated for (1) primary large (>10 mm in anteroposterior and/or mediolateral diameter and/or depth) OLTs not amenable to fixation and (2) nonprimary OLTs.^8^ All patients were required to complete ≥6 months of nonoperative management before the TOPIC procedure. Inclusion and exclusion criteria can be found in Table 1.

**Table 1:** Inclusion and Exclusion Criteria*^*a*^*

Inclusion Criteria	Exclusion Criteria
Patients with a symptomatic OLT (independent of location) who underwent the TOPIC procedure with prospective follow-up	Hemicap and/or arthrodesis after TOPIC
Follow-up >24 mo postoperatively	Follow-up <24 mo
Patients participating in any form of sport preinjury (preinjury AAS >3)	Non−sport related injuries that prevented return to sport (eg, occupational incidents, traffic accidents)

a AAS, Ankle Activity Scale; OLT, osteochondral lesion of the talus; TOPIC, Talar OsteoPeriostic Grafting from the Iliac Crest.

### Surgical Technique and Postoperative Management

All procedures were performed by 2 fellowship-trained foot and ankle orthopaedic surgeons (G.M.M.J.K., S.A.S.S.). The TOPIC procedure completely replaced the osteochondral unit with an autograft including its overlying periosteal layer from the ipsilateral iliac crest. Depending on the location of the OLT, either a medial distal tibial osteotomy or an anterolateral arthrotomy was performed. Thereafter, the OLT was inspected and excised in total; the underlying subchondral bone base was microdrilled. The graft was harvested from the ipsilateral iliac crest (donor site) and personally customized based on the preoperative computed tomography (CT) scan, after which the graft was inserted in a press-fit manner. The operative technique and postoperative management were previously more extensively described for medial and lateral lesions.^7,15^ In case of lateral ankle instability, a lateral stabilization procedure was added.

### Clinical and Sports Evaluation

**Figure 1.**
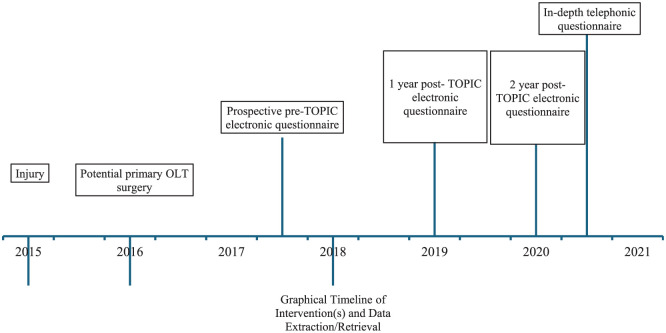
Example of a graphic timeline of interventions and moments of data extraction for 1 specific patient. OLT, osteochondral lesion of the talus; TOPIC, Talar OsteoPeriostic Grafting from the Iliac Crest.

Preoperative patient demographics were extracted from the electronic patient files. At 3 points in time (preoperatively and 1 and 2 years postoperatively), an electronic questionnaire including type, level, hours of sport, and Numeric Rating Scale (NRS) from 0 to 10^27^ of pain during running was filled in by all participants. To verify and elaborate on sport information, a standardized in-depth telephone qualitative and quantitative interview was performed after the 2-year follow-up. The standardized qualitative interview format can be found in Appendix 1. A graphic fictitious timeline of surgical interventions as well as the data extraction moments illustrating the methodology of this study can be found in Figure 1. None of the treating surgeons involved in patient care collected or assessed data to avoid bias.

Information gathered from the electronic questionnaire and interview was used to determine the RTS rates and Ankle Activity Scale (AAS).^11^ In case of a conflict between the electronic questionnaire and the interview information, the interview information was considered determinative. In this study, a modified version of the Ardern RTS criteria was used.^1^ The definitions of the modified (return to/participation in) sports outcomes that were used in the present study can be found in Table 2. The primary outcome of this study was defined as the rate of 2-year postoperative return to any level of sports. The AAS is an ankle-specific standardized activity scoring system that includes 4 general activities, 3 working activities, and 53 sports. A score of 0 points indicates the lowest possible ankle activity, and a score of 10 indicates the highest possible ankle activity. Sports can be categorized as low impact (AAS, 3-4), medium impact (AAS, 5- 7), and high impact (AAS, 8-10). Last, prospectively collected information on complications and additional surgeries was extracted from electronic patient files.

**Table 2:** Definitions of Return to Sports / Sports Participation Outcomes*^*a*^*

Return-to-Sport Category	Definition
Primary outcome: Postoperative participation rate at the patient's desired level of sports, %	The postoperative, self-defined level of sport the patient wishes to achieve following the rehabilitation from the TOPIC procedure; the attainment of this outcome was collectively determined by the participant and researcher during the postoperative interview
Return to any level of sports rate, %	The return to any level of sport (and/or participation in rehabilitation or modified training) of any type and intensity
Return to preoperative level of sport rate, %	The return to the same level of sport as the preoperative situation
Return to preinjury level of sport rate, %	The return to the same level of sport as the preinjury situation
Return to performance rate, %	The return to a higher level of sport compared with the preinjury situation
Participation rate at improved preoperative level of sports, %	The participation in a higher level of sport compared with the preoperative situation

a TOPIC, Talar OsteoPeriostic Grafting from the Iliac Crest.

### Radiological Evaluation

Baseline radiological assessments of the OLT on CT scans included the maximal anteroposterior and mediolateral diameters as well as the lesion depth. The surface area was calculated using the ellipsoid formula (area = coronal length × sagittal length × 0.79), and the volume was determined using the formula volume = coronal length × sagittal length × depth.^2^ The morphological description of the OLT consisted of 3 categories (fragmentary, cystic, or crater).^22^ The preoperative osteoarthritis (OA) stage was determined using an OA grading atlas for CT.^3^

### Statistical Analysis

All statistical analyses were performed with SPSS Version 28.0 (IBM). In cases of a normal distribution of continuous variables, outcomes were reported as mean and standard deviation, and in cases of nonnormal distribution of continuous variables, outcomes were reported as median and interquartile range. Categorical and dichotomous outcome variables were described as absolute number and percentage. To compare preoperative and postoperative data, a paired *t* test was used in cases of normally distributed data, and a Wilcoxon signed-rank test was used in cases of nonnormally distributed data.

Interrater and intrarater reliability for the lesion size and lesion morphology were determined with a subset of 20 OLTs assessed by 2 independent raters (J.D., E.J.G.). Intrarater reliability was performed by repeating the evaluations 2 weeks later. With the use of intraclass correlation coefficient (ICC), lesion size measurements were evaluated.^20^ To test interrater reliability for lesion morphology, the Cohen kappa value was used.^17^ A consensus meeting between the 2 independent raters was organized to agree upon lesion localization and morphological category. A Mann-Whitney *U* test was used to compare the lesion sizes between the primary and nonprimary subgroup in case of nonnormally distributed data. A chi-square test was used to compare the morphology, OA stage, and RTS rates between the primary and nonprimary subgroup. A Wilcoxon signed-rank test was used to compare the preoperative with the 1- and 2-year postoperative AAS and NRS.

In this study, the influence of prognostic factors on the primary outcome has been determined. Because the study group contained 61 participants, 6 factors were chosen: lesion size (surface area), lesion location, lesion morphology, age, preoperative osteoarthritis grading, and body mass index (BMI).^25^ In case of continuous data, a Pearson rho was performed for normally distributed data and a Spearman rho for a nonnormal distribution. A logistic regression was performed for binary data, a multinomial regression for categorical data, and an ordinal logistic regression for ordinal data. The level of significance was set at *P* < .05. A formal sample size calculation was not conducted before the study. Instead, we employed a convenience sampling approach by including all available and eligible patients within the defined time frame.

## Results

### Patient Selection and Demographics

**Figure 2.**
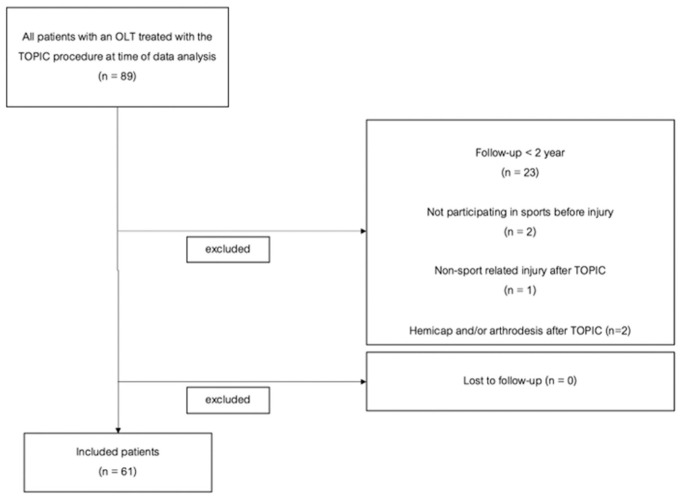
Flowchart of the inclusion process. OLT, osteochondral lesion of the talus; TOPIC, Talar OsteoPeriostic Grafting from the Iliac Crest.

All (100%) of the eligible 61 patients were assessed in the current study (Figure 2). Patient demographics can be found in Table 3, and preoperative lesion characteristics are summarized in Table 4. ICC values of the preoperative lesion size, morphology, and OA rating were determined: 5 were “good” and 1 was at the top of “moderate.” ICC values can be found in Appendix 2 (Table A1). A subgroup comparison of the patient and preoperative lesion characteristics of the primary and nonprimary groups can be found in Appendix 2 (Table A2).

**Table 3:** Preoperative Patient Demographics (N = 61)*^*a*^*

Factor	Value
Patient age, y	31 [25-44]
BMI, kg/m^2^	25 [22-28]
Follow-up, mo	59 [45-67]
Ankle, right/left	30 (49) / 31 (51)
Smoking, yes/no	8 (13) / 53 (87)
Concomitant lateral ankle instability yes/no	11 (18) / 50 (82)
Sex, male/female	24 (39) / 37 (61)
Concomitant procedures performed during TOPIC	Total: 6 (10) (lateral ligament stabilization procedure)
Primary (no previous surgery to the OLT) / nonprimary lesion	23 (38) / 38*^*b*^* (62)
Previous surgeries, n	
0	23
1	10
2	17
3	2
Previous operations for OLT, n	43
Previous operations for osteochondral lesion per patient, mean	0.8

a Data are presented as n (%) or median [IQR] unless otherwise indicated. BMI, body mass index; OLT, osteochondral lesion of the talus; TOPIC, Talar OsteoPeriostic Grafting from the Iliac Crest.

b A total of 22 patients with 1 previous surgery for the OLT and 16 patients with >1 previous surgeries for the OLT.

**Table 4:** Preoperative Lesion Characteristics (N = 61)*^*a*^*

Factor	Value
Lesion size	
Anteroposterior diameter, mm	17 [14-19]
Mediolateral diameter, mm	10 [8-12]
Depth, mm	7 [6-12]
Surface area, mm^2^	159 [127-223]
Volume, cm^3^	1.2 [0.8-2.2]
Lesion location	
Medial	54 (89)
Lateral	7 (11)
Lesion morphology	
Crater	12 (20)
Cystic	38 (62)
Fragmentary	11 (18)
Preoperative ankle osteoarthritis stage^29^	1 [1-1]
Patient stage	
0	7 (11)
1	41 (67)
2	13 (21)
3	0 (0)
Preoperative osteophytes	
Patients	53 (87)
Total osteophytes, n	97
Osteophytes per patient	2 [1-2]

a Data are presented as n (%) or median [IQR] unless otherwise indicated.

### RTS Outcomes

1 year and 2 years after TOPIC, 59% and 82% of patients, respectively, participated in their desired level of sport. Furthermore, 93% and 98% of patients returned to any level of sport 1 year and 2 years, respectively, after TOPIC. All of the modified RTS rates can be found in Table 5. No significant difference in any of the (return to/participation in) sports rates was found between the primary and nonprimary subgroups at both 1 and 2 years of follow-up (*P* = .60 and *P* = .20, respectively). Analysis of prognostic factors showed no significant influence of lesion size, lesion location, lesion morphology, age, preoperative osteoarthritis grading, and BMI on the desired level of sport at both 1 and 2 years after TOPIC; these results can be found in Appendix 2 (Table A3). Sport participation and level of sport preinjury, preoperatively, and 1 and 2 years after TOPIC can be found in Appendix 2 (Table A4).

**Table 5:** Return to Sport Rates and Subgroup Comparison*^*a*^*

	1 Year				2 Years			
	Total (N = 61)	Primary (n = 23)	Nonprimary (n = 38)	*P^*b*^*	Total (N = 61)	Primary (n = 23)	Nonprimary (n = 38)	*P^*b*^*
Postoperative participation rate at desired level of sport (primary outcome)	59 (36/61)	61 (14/23)	58 (22/38)	.82	82 (50/61)	78 (18/23)	84 (32/38)	.56
Return to any level of sports rate	93 (57/61)	91 (21/23)	95 (36/38)	.60	98 (60/61)	96 (22/23)	100 (38/38)	.20
Return to preoperative level of sport rate (only 30 patients participated in sports preoperatively)	83 (25/30)	80 (8/10)	85 (17/20)	.44	93 (28/30)	90 (9/10)	95 (19/20)	.41
Return to preinjury level of sport rate	44 (27/61)	43 (10/23)	45 (17/38)	.92	59 (36/61)	61 (14/23)	58 (22/38)	.82
Return to performance rate	2 (1/71)	4 (1/23)	0 (0)	.20	3 (2/61)	9 (2/23)	0 (0/38)	.09
Participation rate at improved preoperative level of sports	75 (46/75)	73 (17/23)	76 (29/38)	.83	90 (55/61)	78 (18/23)	97 (37/38)	.02

a Data are presented as a percentage (ratio).

b Comparison of primary versus nonprimary.

### Clinical Sport Outcomes

**Figure 3.**
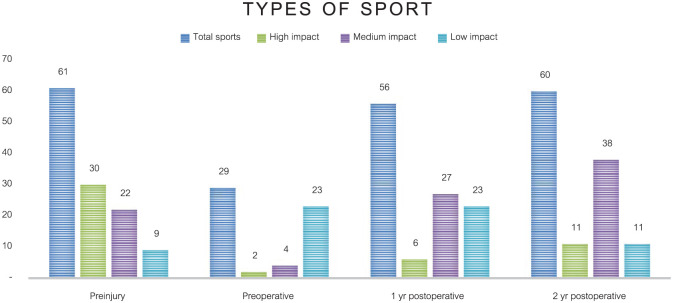
Distribution of sports impact level over time. High impact: AAS of 8, 9, and 10; medium impact: AAS of 5, 6 and 7; low impact: AAS of 3 and 4. AAS, Ankle Activity Score.

The AAS increased significantly from 2 points preoperatively to 5 points at both 1 and 2 years after TOPIC (*P* < .05). The NRS during running improved significantly from 9 to 5 points both 1 and 2 years after TOPIC (*P* < .05). AAS and NRS scores for the preinjury, preoperative, 1 year, and 2 years after TOPIC are shown in Table 6. A graphic distribution of patients participating in high-, medium-, and low-impact sports preinjury, preoperatively, 1 year after TOPIC, and 2 years after TOPIC can be found in Figure 3.

**Table 6:** Ankle Activity Scale (AAS) and Numeric Rating Scale (NRS) for Pain Pre- and Postoperatively*^*a*^*

	Preinjury	Preoperative	1 Year	*P^*b*^*	2 Years	*P^*b*^*
AAS	7 [5-8]	2 [2-3]	5 [3-5]	<.01	5 [5-6.5]	<.01
NRS Running	NA	9 [8-10]	5 [1-8]	<.01	5 [1-8]	<.01

a Data are presented as median [IQR]. NA, not applicable.

b Comparison preoperative with 1 or 2 years postoperatively.

### Complications and Additional Surgeries

In this cohort, 2 complications (3%) were encountered; 1 case of transient hypoesthesia surrounding the iliac crest scar and 1 case of transient hypoesthesia of the first web space. Both complications resolved without surgery and did not limit the rehabilitation process or the ability to participate in sports 1 and 2 years after TOPIC. No patients experienced persistent donor-site morbidity 2 years postoperatively. A total of 33 additional surgeries on 23 patients were performed with a mean interval of 14.2 months between TOPIC and the additional surgical procedure. Only 10% of additional procedures were related to the graft; the main causes of reoperation were symptomatic hardware and ankle impingement (76% combined). All information on the additionally performed surgical procedures after TOPIC can be found in Appendix 2 (Table A5).

## Discussion

The primary finding of the present study is that the TOPIC procedure for large, complex OLTs results in 8 out of 10 patients participating at their desired level of sport 2 years after surgery. This study also found that almost all patients returned to their sport at any level and 6 out of 10 returned to their preinjury level of sport 2 years postoperatively. The AAS significantly increased from 2 preoperatively to 5 and the NRS for pain during running improved significantly from 9 to 5 points at 2-year follow-up. Despite significant improvement in pain during running, the mean postoperative NRS remained 5, indicating that moderate pain persisted in some patients. This may be related to symptomatic hardware still in place or suboptimal rehabilitation. A standardized rehabilitation protocol supervised by an experienced physical therapist focused on balance, strength, and a phase-based RTS progression may help to reduce pain. The TOPIC procedure enabled most patients to RTS with high goal attainment, reinforcing its clinical value and supporting informed shared decision making.

When evaluating RTS outcomes after osteochondral autograft procedures for large OLTs, a systematic review found that 90% of patients returned to any level of sport after ≥24 months follow-up.^24^ These results can be considered comparable with those found in the present study. With respect to return to preinjury level of sport, the previously mentioned systematic review found that osteochondral autograft procedures show a slightly higher return to preinjury level of sport rate of 72% compared with the 59% observed in the present study. We hypothesize that this difference may be due to differences in patient selection; the TOPIC procedure can be regarded as a “salvage procedure” because of the challenging nature of the treated OLTs (large, mostly nonprimary, challenging morphology, and relatively high OA scores).^21^

When considering change in level of sport participation, and AAS, patients tended to return to lower-impact sport activities. This is in line with Paul et al^19^ who observed changes in the type and level of sport, with fewer patients participating in high-impact and contact sports. Moreover, the aforementioned authors found that the AAS in 130 OLT cases treated with osteochondral transplantation decreased from 5.9 preinjury to 5.0 postoperatively. The activity rating scale^18^ decreased from 8.9 preoperatively to 6.8 postoperatively. It can be hypothesized that both physical restrictions (ie, residual pain, postoperative restrictions in range of motion or function) and mental restrictions (ie, fear of reinjury, pain catastrophizing, unwillingness to return to previous sport) may contribute to the RTS and level of participation, as has previously been suggested in other orthopaedic literature such as anterior cruciate ligament reconstruction patients.^30^ Moreover, when considering the rate of participation at the desired level of sport, it can be concluded that such factors already influence participation before surgery, potentially affecting postoperative results. It is therefore important to optimally counsel patients and monitor physical and mental well-being both in the preoperative setting and during the rehabilitation phase to obtain optimal outcomes.

Although a relatively high reoperation rate was observed in the present study, previous findings at the 1-year postoperative time point indicated high patient-reported outcomes following the TOPIC procedure. This suggests that reoperations may have a limited impact on midterm functional and sports outcomes. Therefore, while efforts to reduce the reoperation rate, such as enhancing preoperative assessments, standardizing postoperative rehabilitation, and improving patient education, are important, the overall functional and sports outcomes may remain favorable even in cases requiring reoperation.

### Strengths and Limitations

The strengths of this study include the 100% follow-up rate, the prospective nature of the study, and the use of independent assessors for radiological data collection from CT scans with high intra- and interrater reliability. An additional strength is the use of both quantitative and qualitative data collected from the telephonic interviews, during which the prospectively collected data were verified and double-checked. These interviews included a full reconstruction of the patients’ course of disease with respect to their OLT and therefore provide a clinically representative view of the (return to/participation in) sport outcomes. Another strength is the use of a newly developed outcome—namely, the desired level of postoperative RTS. This method of scoring RTS involves a self-defined, personalized goal set by the patient, functioning as goal attainment scaling (GAS). GAS is a tool that ensures a holistic, patient-centered evaluation, and it is a valuable component in patient care, facilitating a more individualized, engaging, and effective treatment process.^12,13^ On the other hand, the desired level of sport outcome is more subjective compared with the other sport outcomes, and potential biases could occur.

The present study is not without limitations. First, the study does not include a control group. Second, no formal power calculation was performed; we aimed to include the largest number of patients through a prospective convenience sample approach, resulting in low-power comparative analyses. Additionally, no correlation was found between preoperative factors and RTS outcomes, which may be related to the study size. Last, this study included a heterogeneous study group as the postoperative rehabilitation quality, age (preinjury and preoperative) level of sport, lesion size and morphology, osteoarthritic grading of patients, and BMI varied greatly. A previous study found that high BMI was associated with higher patient-reported pain outcomes,^8^ and another study found that BMI was not significantly correlated with the reported activity levels of patients treated with osteochondral transplantation of the talus.^19^ Additional limitations include the inclusion of only active patients, which limits the generalizability of the findings to less active populations. Furthermore, patients who received an ankle fusion after TOPIC were excluded, possibly leading to an overestimation of outcomes. Last, a few patients underwent a concurrent lateral ankle stabilization procedure; the limited sample size prevented subgroup analyses to evaluate the influence of this intervention.

### Future Directions

In this study, BMI, as well as the other assessed prognostic factors, showed no significant influence on the return to desired level of sport 1 and 2 years after TOPIC—thus, this needs to be further explored in the future. Additionally, long-term sport-specific follow-up after the TOPIC procedure in a larger study population with a subgroup analysis on the influence of concomitant lateral ankle stabilization is suggested.

This study is clinically relevant, as it enables more in-depth counseling of patients undergoing autologous osteoperiostic grafting for large OLTs specifically on postoperative sport outcomes, thereby enabling a more substantiated shared decision-making and improving expectation management. From a research perspective, the novel methodological framework with the use of in-depth qualitative telephone interview and modified definitions of RTS and participation in sport may help to improve understanding of return to/participation in sport outcomes after cartilage surgery of the ankle. This is important, as RTS is a complex outcome measure influenced by multiple variables, but it is highly important for patients.

## Conclusion

At 2 years of follow-up, almost all patients were able to RTS after the TOPIC procedure for large OLTs. Eight out of 10 patients resumed participation at their self-reported desired level of sport, and 6 out of 10 even returned to their preinjury level of sport. Ankle activity improved significantly and pain while running decreased significantly. Despite these positive functional outcomes, 1 in 3 patients required additional surgery (mostly hardware removal) within 2 years. Overall, this joint-sparing procedure provides durable improvement in sports participation and function. The findings of the present study may assist patients and physicians in a more informed, shared decision-making process.

## Appendix 1

### Standardized Qualitative Interview Format

#### Preoperative Characteristics

- According to the online questionnaire, you played … right before your surgery, is that right? Did you play sports right before your surgery?
- At what level did you practice this sport (top level, competitive, recreational)? Can you specify this?
  ○ Top level/professional (Olympic Games, World Championships, European Championships, World Cups, National team, National championships at the highest level, paid sport)
  ○ Competitive (soccer/hockey/rugby competition on the third league or lower, national championships on any level lower than elite sports)
  ○ Recreative (50+ hours of sport annually)

- How many hours per week did you train right before surgery?
- Did you do another sport/activity right before your surgery?

#### Postoperative Characteristics

- According to the online questionnaire, 1 year after your surgery you played … is that correct? Did you play sports 1 year after surgery?
- At what level did you practice this sport (top level, competitive, recreational)? Can you specify this?
  ○ Top level/professional (Olympic Games, World Championships, European Championships, World Cups, National team, National championships at the highest level, paid sport)
  ○ Competitive (soccer/hockey/rugby competition on the third league or lower, national championships on any level lower than elite sports)
  ○ Recreative (50+ hours of sport annually)

- How many hours did you train 1 year after the surgery?
- Did you play another sport or do another physical activity 1 year after surgery?
- If yes: how many hours per week did you do this activity?
- Before undergoing the surgery, what were your expectations regarding returning to sports after recovery?
- Did you meet these expectations?
- How did your sport/physical activity ability develop from 1 year after surgery until now?

## Appendix 2

**Table A1:** Table Interrater and Intrarater Reliability for the Lesion Size and Lesion Morphology*^*a*^*

	Interrater Reliability	Intrarater Reliability
Lesion size	0.86	0.89
Lesion morphology	0.71	0.82
OA grading	0.80	0.83

a OA, osteoarthritis.

**Table A2:** Preoperative Patient and Lesion Characteristics Subgroup Comparison*^*a*^*

	Primary (n = 23)	Nonprimary (n = 38)	*P*
Patient age, y	31 [25-40]	33 [24-50]	.51
BMI, kg/m^2^	25.9 [22.0-29.6]	24.9 [22.4-27.3]	.80
Smoker, yes/no	4 (17) / 19 (83)	4 (11) / 34 (89)	.99
Ankle instability, yes/no	4 (17) / 19 (83)	7 (18) / 31 (82)	.92
Size			
Depth, mm	9 [6.0-14.5]	7 [6-10]	.26
AP diameter, mm	18 [13-21]	16 [14-18]	.14
Lesion surface, mm^2^	175 [109-269]	157 [128-200]	.37
ML diameter, mm	10 [8-13]	10 [8-12]	.52
Lesion volume, cm^3^	1.2 [0.9-3.5]	1.1 [0.8-1.8]	.25
Morphology			
Crater lesion	2 (9)	10 (26)	.31
Cystic lesion	16 (70)	22 (58)	.36
Fragmentary lesion	5 (22)	4 (11)	.09
Hemicap	0 (0)	2 (5)	.28
Press fit/non−press fit TOPIC procedure			
Press fit	21 (91)	31 (82)	.30

a Data are presented as median [IQR] or n (%). AP, anteroposterior; BMI, body mass index; ML, mediolateral; TOPIC, Talar OsteoPeriostic Grafting from the Iliac Crest.

**Table A3:** Prognostic Factors 1 and 2 Years After TOPIC*^*a*^*

	Test	1 Year Postoperatively	2 Years Postoperatively
Lesion size	Spearman rho	.67	.96
Lesion location	Logistic regression	.15	.77
Lesion morphology	Multinominal regression	.66	.70
Age, y	Spearman rho	.39	.26
Preoperative osteoarthritis grading	Ordinal logistic regression	.09	.08
BMI, kg/m^2^	Pearson rho	.22	.38

a Data are presented as *P* values. BMI, body mass index; TOPIC, Talar OsteoPeriostic Grafting from the Iliac Crest.

**Table A4:** Sport Participation and Level of Sport Preinjury, Preoperatively, and 1 and 2 Years After TOPIC*^*a*^*

	Preinjury	Preoperatively	1 Year Postoperatively	2 Years Postoperatively
Total sports participation	61 (100)	29 (48)	57 (93)	60 (98)
Recreational level	31 (51)	27 (44)	52 (85)	55 (90)
Competitive level	30 (49)	2 (3)	3 (5)	4 (7)
Professional level	0 (0)	0 (0)	1 (2)	1 (2)
No sports	0 (0)	32 (52)	5 (8)	1 (2)

a Data are presented as n (%). TOPIC, Talar OsteoPeriostic Grafting from the Iliac Crest.

**Table A5:** Additional Surgical Procedures After TOPIC*^*a*^*

	Value
Patients requiring additional surgery	23 (38)
Additional surgeries	33
Additional subprocedures performed during additional operations	49
Procedures related to graft*^*b*^*	5 (10)
Arthroscopic shaping/reduction of graft	2 (4)
Arthroscopic shaping of graft and debridement/BMS of graft	2 (4)
Open shaping/reduction of graft	1 (2)
Procedures unrelated to graft*^*b*^*	44 (90)
Implant removal for discomfort at osteotomy site	22 (45)
Arthroscopic debridement for ankle impingement	15 (31)
Open debridement for ankle impingement	2 (4)
Open and arthroscopic debridement for ankle impingement	2 (4)
Other procedures	3 (6)

a Data are presented as n or n (%). BMS, bone marrow stimulation; TOPIC, Talar OsteoPeriostic Grafting from the Iliac Crest.

b Values are presented as n (%) of total number of additional procedures performed during additional operations.
